# Stimulation, Recording Potential and Antimicrobial Medical Catheter Coatings

**DOI:** 10.1155/MBD.1994.445

**Published:** 1994

**Authors:** Richard B. Beard, Mark DeLaurent, Kambiz Pourrezaei, Sorin Adrian

**Affiliations:** 1 Electrical and Computer Eng. - Biomedical Eng. and Science Institute Drexel University PA 19104 USA; 2 Memorial Hospital Department of Radiology Easton MD USA; 3 Siemens Medical System PA USA

## Abstract

Biocompatibility of electrodes for stimulation are difficult to maintain homeostasis. Noble metal stimulating electrodes which are normally biocompatible on keratinized tissue become very non-biocompatible when they are interfaced with nonkeratinized tissue in an area such as the oral cavity. Composite electrodes have been made biocompatible in the oral cavity even at current densities larger than 1 μA/mm^2^. Electrodes used in potential readings require that the anodic and cathodic polarization remain minimal. Silver-silver chloride electrodes are minimal. Silver-silver chloride electrodes are not always reversible. The range of pH, voltages and current densities when silver-silver chloride are not reversible are presented. Recently at Drexel University reliable silver coatings inside and outside of medical catheters have been fabricated to act as antimicrobial to a variety of bacteria. Noble and nonnoble metals have been combined in coatings with silver to enhance the antimicrobial action.


### STIMULATION, RECORDING POTENTIAL AND ANTIMICROBIAL MEDICAL CATHETER COATINGS

Richard B. Beard[1], Mark DeLaurent[2], Kambiz Pourrezaei[1], and Sorin Adrian[3]

[1] Electrical and Computer Eng. - Biomedical Eng. and Science Institute, Drexel University, PA 19104, USA
[2] Memorial Hospital, Departrnent of Radiology, Easton, MD, USA
[3] Siemens Medical System, PA , USA

Abstract

Biocompatibility of electrodes for stimulation are difficult to maintain homeostasis. Noble metal stimulating electrodes which are normally biocompatible on keratinized tissue become very non-biocompatible when they are interfaced with nonkeratinized tissue in an area such as the oral cavity. Composite electrodes have been made biocompatible in the oral cavity even at current densities larger than 1 $\mu A/mm^2$. Electrodes used in potential readings require that the anodic and cathodic polarization remain minimal. Silver-silver chloride electrodes are minimal. Silver-silver chloride electrodes are not always reversible. The range of pH, voltages and current densities when silver-silver chloride are not reversible are presented. Recently at Drexel University reliable silver coatings inside and outside of medical catheters have been fabricated to act as antimicrobial to a variety of bacteria. Noble and nonnoble metals have been combined in coatings with silver to enhance the antimicrobial action.

### I Introduction

The following article discusses the use of silver in three diversified biomedical applications at Drexel University. The first application considers the biocompatibility problems with noble metal stimulating electrodes at the gingiva interface in a moist oral cavity[1]. The second application considers the parameters which affect the silver-silver chloride reference electrode used in physiological potential measurements [9, 10]. The third application considers the use of silver in the development of antimicrobial antothrombogenic coatings for inside and outside of medical catheters [24, 25, 26].

### II Development of Biomedical Electrode for D.C. Stimulation in the Oral Cavity.

The successful use of stimulating electrodes in a biomedical application must address the importance of electrode geometry and material as well as the site of stimulation in the body [1]. D.C. electrodes were applied to the gingiva to produce D.C.. electric currents for possible increase in alveolar bone osteoblasts and enhanced cellular enzymatic phosphorylation activities in peridontal tissues [2,3]. The original electrode materials when applied to the human gingiva interface produced severe irritation. At this time co-operative research between Drexel University and the University of Pennsylvania School of Dental Medicine was undertaken to develop biocompatible electrodes capable of handling direct currents exceeding 20μA/cm$^2$. The gingiva and epithelium of the cheek lining are moist with saliva and only partially keratinized unlike the tough keratinized body surface in contact with dry air. Figure 1 illustrates the marked initiation surrounding noble metal electrodes when used for d.c. stimulation in the oral cavity of the cat.

severe irritation

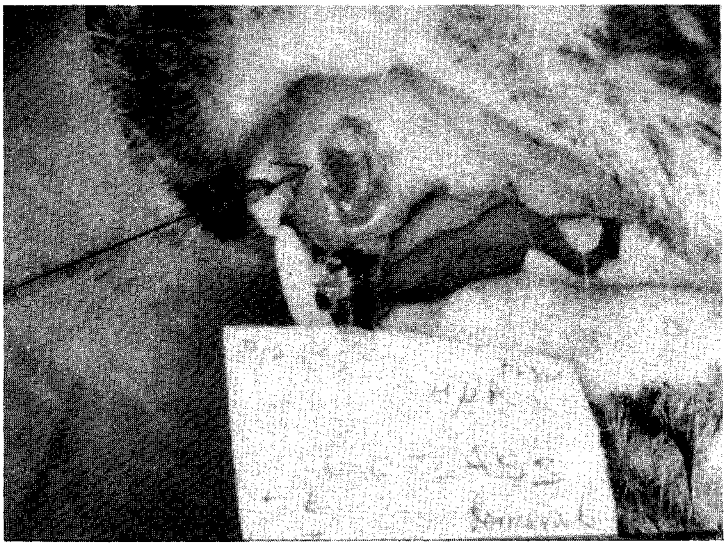

Figure 1 Severe initiation produced at a gold-electrode interface of a cat after stimulation with a direct current of 20 μA/cm$^2$.

A biocompatible composite electrode was developed which was capable of operating in the Oral cavity with currents well above 20μA/cm$^2$ with excellent biocompatibility [1, 4].. The composite electrode consisted of conductive gels at the gingiva interface to remove d. c. stimulating electrodes from the moist tissue. Passage of current across the cathode shifted the pH towards 8.0 due to the production of $OH^-$ ions, which produced redness at the gingiva interface. In order to shift the pH out of the basic region, lemon juice or citric acid was added to cathode gel consisting of 0.0154 M KCl in agarous gel.

Silver cathodes placed behind the cathode gel discolored the gingival-gel interface due to the silver ion migration through the gel. Figure 2 shows this discoloration at the cathode for a silver anode and cathode placed in cathodic gel for 48 hours with a large current density of 400 μA/cm$^2$ at the electrodes.

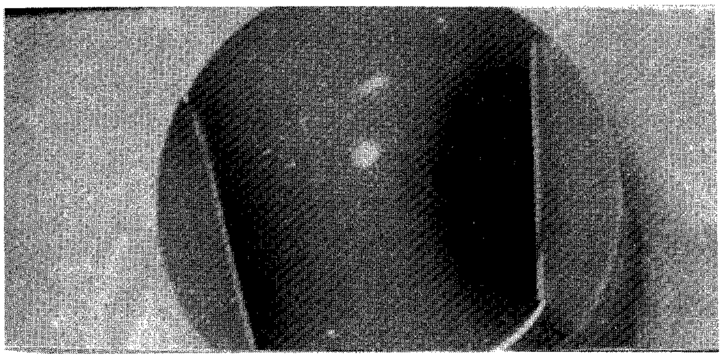

**silver anode** **silver cathode**

Figure 2 A Silver anode and cathode placed in a container of cathode gel consisting of seaweed agarose gel with a 2mg/100 ml concentration, 0.0154M KCl and citric acid. A current density of 400 $\mu A/cm^2$ was placed across each electrode for 48 hours at room temperature.

The same problem of ion migration has been encountered in fabricating hybrid circuits with gold or silver. Platinum-silver and platinum-gold fired as a paste onto aluminum ceramic substrates solved the migration problems in thick film technology, [5]. This same technique used to form platinum silver alloy electrodes solved the migration problem for direct current application in the oral cavity. The migration at the platinum-silver alloy cathode was far less than for a pure silver cathode. The silver-anode was searated via a salt bridge from the cathode for silver migration to insure that no anode products would interfere at the cathode. Figure 3 demonstrates that the Platinum-silver cathode gel with a current of 400$\mu A/cm^2$ produces far less migration of silver than the silver electrode of Figure 2.

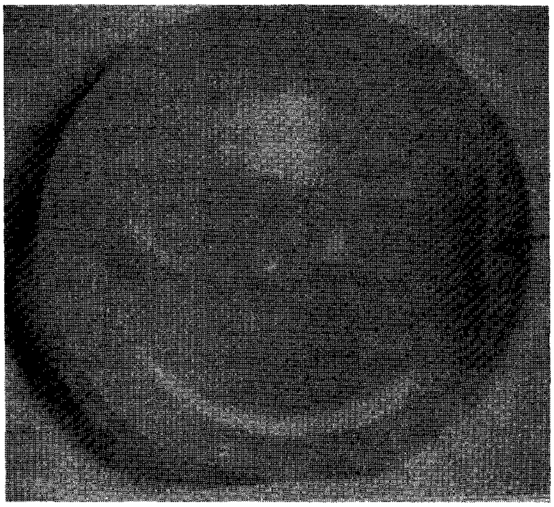

Figure 3 Platinum-silver electrode in cathode gel. Current density 400$\mu A/cm^2$ was maintained for 48 hours across cathode.

After 5 days of current density of 20 $\mu A/cm^2$ at the electrode gingiva interface, the tissue remained similar in appearance to what it was before insertion of the appliance. Histopathological examination of tissue under the composite electrodes showed no signs of inflammation. Toxicity evaluation of the electrode gels by a certified laboratory demonstrated the acceptability of the gels.

After approval by a human evaluation committee the appliances with biocompatible electrodes were placed in the oral cavity of humans. The composite electrodes proved to be biocompatible during research and clinical evaluation.

### III Evaluation of Silver-Silver Chloride Electrode Under Controlled voltage and pH

In biomedical applications the Ag/AgCl electrode has been widely used to monitor electro-physiological potentials which are usually in the microvolt range. Millions of Ag/AgCl disposable ECG electrodes are used in clinical applications each year. The Ag/AgCl as a secondary reference standard is widely used because of it's electrochemically reversible reaction and stability. The disposable electrodes are used with a highly conductive gel containing chloride ions between the silver electrode and keratinized interface. The incorporation of an oxidizing agent into the electrically conductive interface facilitates the production of silver chloride to silver and chloride ions and counteracts the decomposition of silver chloride to silver and chloride ions [7]. Methods other then powder metallurgical compaction have been presented in detail [12].

Solid State Electrodes in the form of silver halide discs have the advantage of ruggedness. There are disadvantages such as the variation of the surface resistivity of silver chloride and variation due to photoelectric effects,[8] Compacts of silver-silver chloride of different compositions were fabricated at Drexel. In the discussion below, the changes in surface resistivity and over potential are considered for different compositions and current densities. Linear A.C. electrode polarization impedance measurements on Ag/AgCl electrodes conducted under controlled pH and potential, demonstrate the sensitivity of the electrode kinetics to these parameters. These results are useful in determining under what conditions Ag/AgCl electrodes will be reliable reference electrodes.

In order to give a basis for the understanding of polarization and impedance graphs presented in this section, a short introduction to the kinetics of electrode processes is presented [13, 14].

When an ion donates an electron to an electrode the ion is oxidized and is the anodic current density. When an ion accepts an electron from the electrode it is reduced and i is the cathodic current density. The kinetic properties of the interface are represented by anodic and cathodic currents. The current-overpotential relationship at the interface can be considered in terms of deviations from the equilibrium potential. The net current at equilibrium will be zero requiring the cathodic and anodic current densities to be equal.

At equilibrium,

$\overleftarrow{i} = \overrightarrow{i} = i_0$ exchange current density anodic current density

$$\overleftarrow{i} = Fk_b C_R e^{\frac{(1-\alpha)F\Delta\phi_e}{RT}}$$

F is the Faraday

$\alpha$ is the transfer coefficient

$k_b$ and $k_f$ rate constants cathodic current density

$$\overrightarrow{i} = Fk_f C_o e^{-\frac{\alpha F\Delta\phi_e}{RT}}$$

$C_R$=concentration of reduced species; $C_O$=concentration of oxidized species

R= molar gas constant; T= absolute temperature

$\Delta\phi_e$=equilibrium potential difference across the electrode

At non-equilibrium

$\overleftarrow{i} - \overrightarrow{i} = i$ Net current

$$i = Fk_b C_R e^{\frac{(1-\alpha)F\eta\Delta\phi}{RT}} - Fk_f C_o e^{-\frac{\alpha F\Delta\phi}{RT}}$$

$\Delta\phi$=nonequilibrium potential difference

The difference between the nonequilibrium and equilibrium potentials is the overpotential, $\eta$

$$\eta = \Delta\Phi - \Delta\Phi_\varepsilon$$

$$i = i_0\left(e^{\frac{(1-\alpha)F\eta}{RT}} - e^{-\frac{\alpha F\eta}{RT}}\right)$$ Butler Vollmer Equation

For small over potential where $|\eta| \leq 0.01\text{V}$ the exponential can be expanded to give

$$i = i_0 \frac{F\eta}{RT}$$

Charge transfer resistance, $R_{CT} = \frac{\eta}{i} = \frac{RT}{\eta F i_0}$

The above discussion forms a basis of evaluating the departure of the electrode potential from the reversible or equilibrium value upon passage of faraday current [13]. This polarization is an indication of the reversibility of the reference electrode Ag/AgCl.

Figure 4 illustrates that a compact which is 10 % Ag and 90 % AgCl has, when the current densities are of the order of 1 $\mu A/cm^2$, large anodic overpotentials which make it a poorly reversible electrode [9].

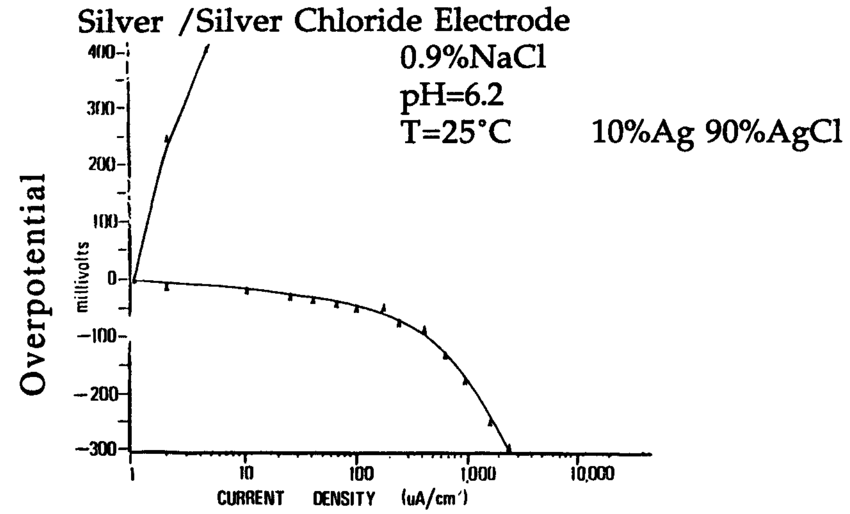

Figure 4 Current Densities for 10%Ag 90%Cl Compact

On the other hand Figure 5 demonstrates that a 90 % Ag and 10 % AgCl has anodically even upto 100 $\mu A/cm^2$, only a small overpotential but cathodically, even at 10 $\mu A/cm^2$, overpotentials which are unacceptable..

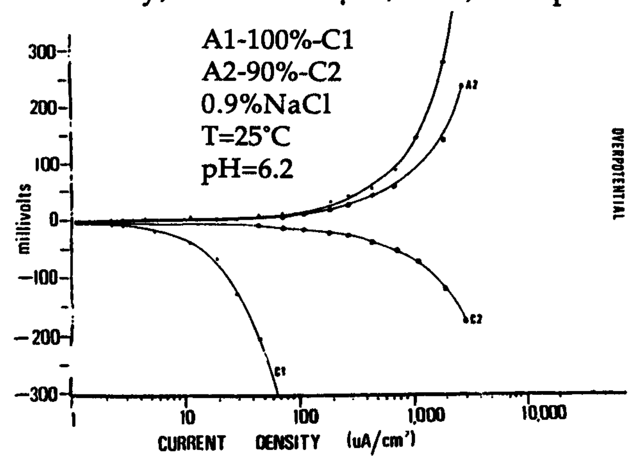

Figure 5

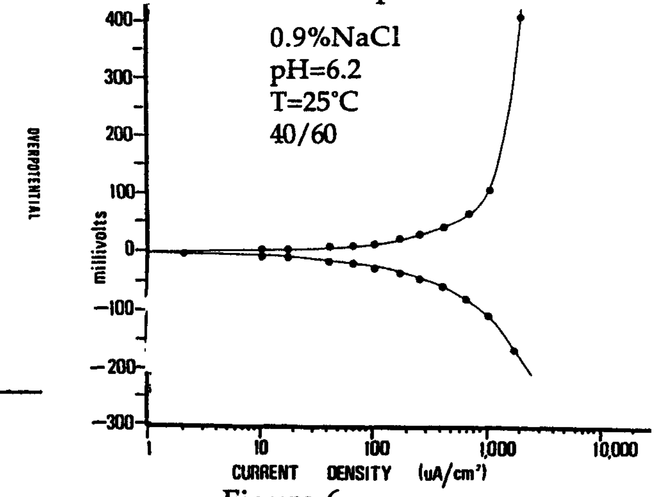

Figure 6

However, when the silver or silver chloride compacts are in the range of 40% Ag 60%AgCl or 60%Ag 40%AgCl the overpotentials are relatively small indicating good reversible electrodes as shown by Figure 6. The overpotentials for various current densities were obtained by using a galvanometer technique with calomel reference electrode,[9].

A four point resistive probe developed for semi-conductor materials [15, 16] was applied to the measurement of the Ag/AgCl compacts, A Veeco's Four Point Probe, FPP-100 was used to measure the resistivities of the fabricated compacts. Silicon wafers of known bulk resistivity were used to calibrate the FPP-100 [9]. Figure 7 demonstrates that the surface resistivities are fairly constant and low between 40 % to 60 % silver which agrees with the above overpotential results.

Below 40 % silver, the resistivity increases dramatically and above 70 % silver it decreases sharply which could account for the polarization outside of the 40 % to 70 % silver range.

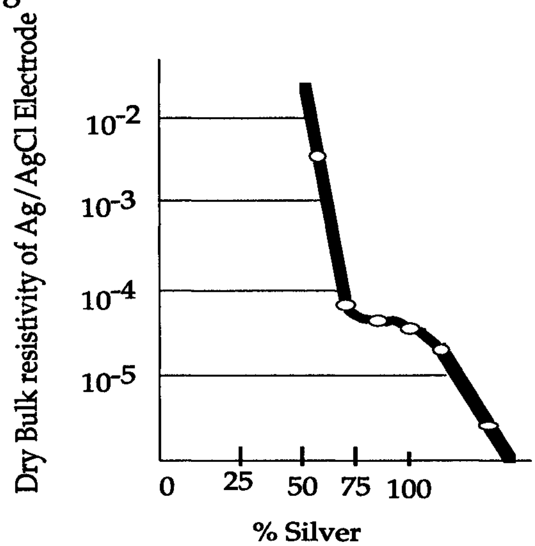

Figure 7 Bulk Resistivity of Ag/AgCl Electrode Vs percent silver in Ag/AgCl compact electrodes.

In the above it was seen that for small overpotentials the relationship between overpotentials and current density can be considered in terms of a charge transfer resistance. The interface under controlled voltage and current can be considered in terms of an electrical impedance to represent other rate determining mechanisms at the interface such as diffusion and adsorption. The potential-pH equilibrium diagram for the silver-water system at 25°C is shown in Figure 8 with constant pH lines of 6, 7 and 8 and constant voltage lines from -0.4 V to +0.8 V. This is the same technique Brownstein used in the evaluation of platinum and palladium electrodes under controlled voltage and pH, [17, 18]

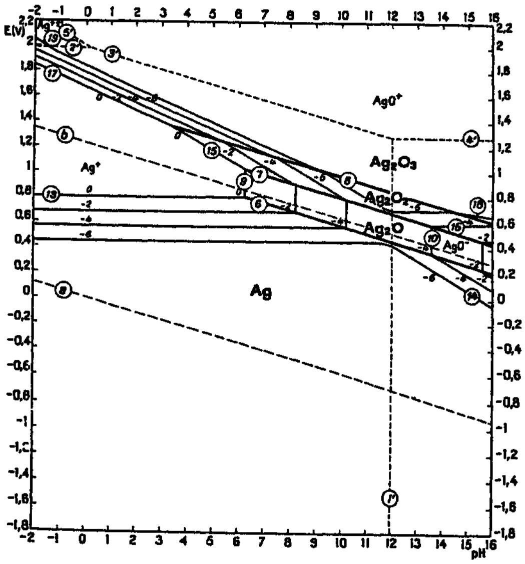

Figure 8 Potential -pH equilibrium diagram for the system Silver-Water at 25°C

The admittance (conductance and capacitance) measurements, of the electrode-electrolyte system at a given pH and controlled potential across the interface were made over a frequency range of 250 to 50000 Hz [10,11]. A Wayne Kerr B221 universal bridge was used with a General radio 1312 decade oscillator as an external source and General Radio 1232-A tuned amplifier-null detector. The glass cell was monitored at a temperature of 25°C.

The electrode-electrolyte was Ag/AgCl in 1 M NaCl and physiological saline. pHs were varied using different concentrations of NaOH and HCl and monitored with an Orion pH meter.

At each pH six different potentials were applied across the interface with a mini-potentiostat, [20]. An electrometer (Keithley 610C) was used to monitor the applied d.c.. potential against a standard calomel reference electrode [17, 20]. The system admittance was measured at each potential and 12 different frequencies from 250 to 50000 Hz. Frequencies were measured on an H.P. 5326B Time counter-DVM. Figure 9 is a graph of the electrode parallel conductance vs. electrode potential for Ag/AgCl Electrode in 1 M NaCl at 25°C and pH=7.1 [10, 11].

Figure 11 is a graph of the electrode parallel capacitance vs electrode potential (vs NHE) for a Ag/AgCl electrode in 1 M NaCl at 25°C, pH=7). Figure 12 is a graph of the polarization impedance in 1 M NaCl at 25°C, pH=7.1. NHE is the normal hydrogen electrode

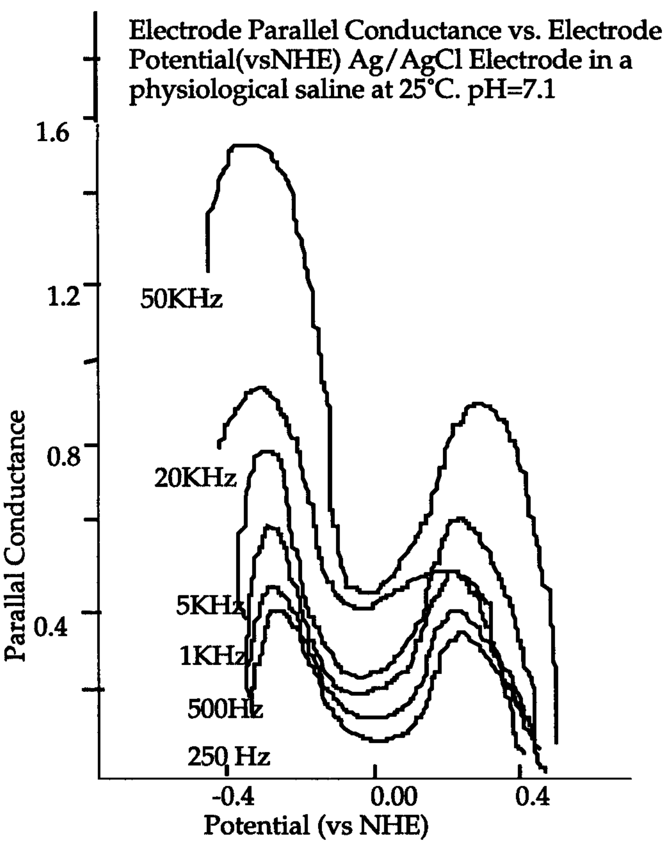

Figure 9. Electrode Parallel Conductance Vs Electrode Potential (Vs NHE) for Ag/AgCl electrode in 1 M NaCl at 25 °C, pH=7.1

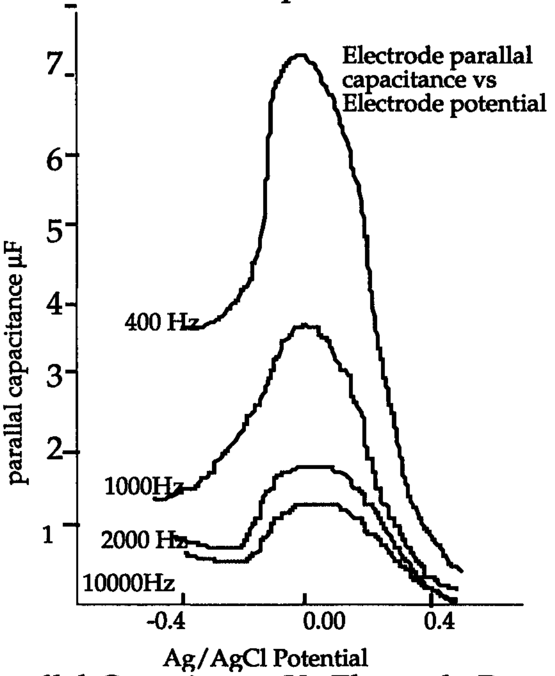

Figure 10. Electrode Parallel Capacitance Vs Electrode Potential (Vs NHE) for Ag/AgCl electrode in 1 M NaCl at 25 °C, pH=7.1

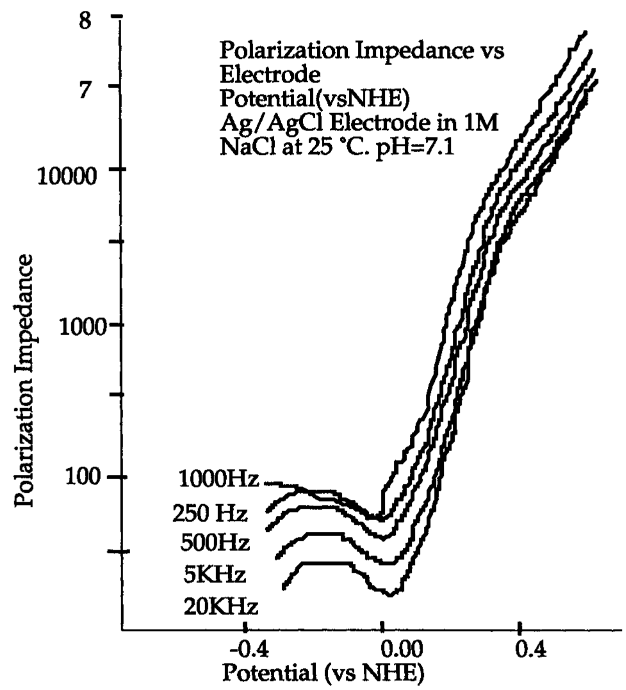

Figure 11 Polarization impedance Vs electrode potential (Vs NHE) for Ag/AgCl electrode in 1 M NaCl at 25 °C, pH=7.1

On observing figure 11 it is noted that a Ag/AgCl electrode in a 1 M NaCl solution at a pH=7.1 has the polarization impedance increase by orders of magnitude at positive potentials. In 1M NaCl solutions the results are similar for Ag/AgCl electrodes in acidic and basic solutions. In physiological saline solutions the Ag/AgCl electrodes for a pH of 6.0 and 7.1 have much greater changes in the polarization impedance on the cathodic or negative potential side.

It is concluded that the electrode polarization impedance for a Ag/AgCl electrode varies markedly for clamped potentials away from the zero potential vs NHE. A Ag/AgCl electrode when improperly used in measurements will not be reversible for large overpotentials and polarization impedances. The large polarization impedance will interfere with biopotentials monitoring and impedance measurements.

### IV Development of Antimicrobial and Antithrombogenic Coatings for the Inside and Outside of Medical Catheters

Catheters uesd as medical devices have the disadvantage of providing a pathway for the colonization and migration of bacteria into the body. Silver has been used as an antibacterial agent in a large variety of medical applications [20, 21, 22]. The first International Conference on Gold and Silver in Medicine [20], devoted a number of papers to the use of silver to inhibit bacteria. In this presentation consideration is given to a technique for coating the interior as well as the exterior surface of Teflon, latex or polyurethane catheter with silver and other metals such as platinum. The evaluations of the coatings have been accompalished in vitro using BHI broth inoculated with E. Coli obtained from a hospitalized patient suffering from urinary catheter related sepsis [24]. In vivo evaluations of catheters were performed using the New Zealand white Rabbit (NZW) [25].

The plating of Ag inside and outside of a catheter entailed the cleaning and preparation of the catheter surface followed by electrode plating with a plating solution of silver nitrate, sodium dodecylbenzene sulfonic acid sodium together with a reducing solution of hydrazine hydrate [23, 26]. Additional silver on the outside surface were coated with a vaccuum arc and cylinderical magnetron [26]. Platinum was deposited over silver using a standard electroplating technique.

The invitro measurements based on turbidity consisted of inoculating 5 ml of liquid broth with $10^4$cfu/ml of a given bacteria and then culturing with given sample at 37°C for 120 hours. The turbidity was monitored by optical density changes [24, 25]. The optical density variation from 0.1 to 1.0 is equivalent to 0 to $10^8$ cfu/ml where cfu=colony forming unit.

Surface of coated sample to liquid volume ratio S/V=1 for control

| Sample | Surface Area | S/V |
|---|---|---|
| a-Control Teflon Catheter | 25 $mm^2$ | 1 |
| b-Ag Coated Teflon | 25 $mm^2$ | 1 |
| c-Ag Coated Teflon | 50 $mm^2$ | 2 |
| d-Ag Coated Teflon | 100 $mm^2$ | 4 |
| e-Ag/Pt Coated Teflon Pt | 25$mm^2$ Ag/25$mm^2$ | |
| f-Ag/Pt Coated Teflon Pt | 50$mm^2$ Ag/50$mm^2$ | |

Refering to the above legend the figures 12 and 13 demonstrate the effect of varying surface area on the growth of E Coli measured by optical density [24,26]

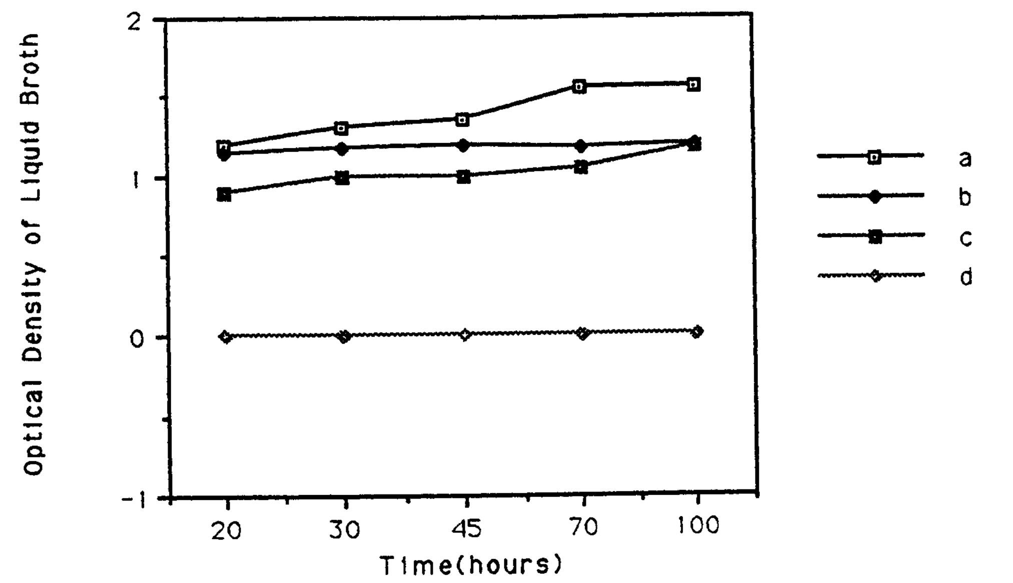

Figure 12 Effect of varying surface area for Ag-Coated catheters Samples a- d

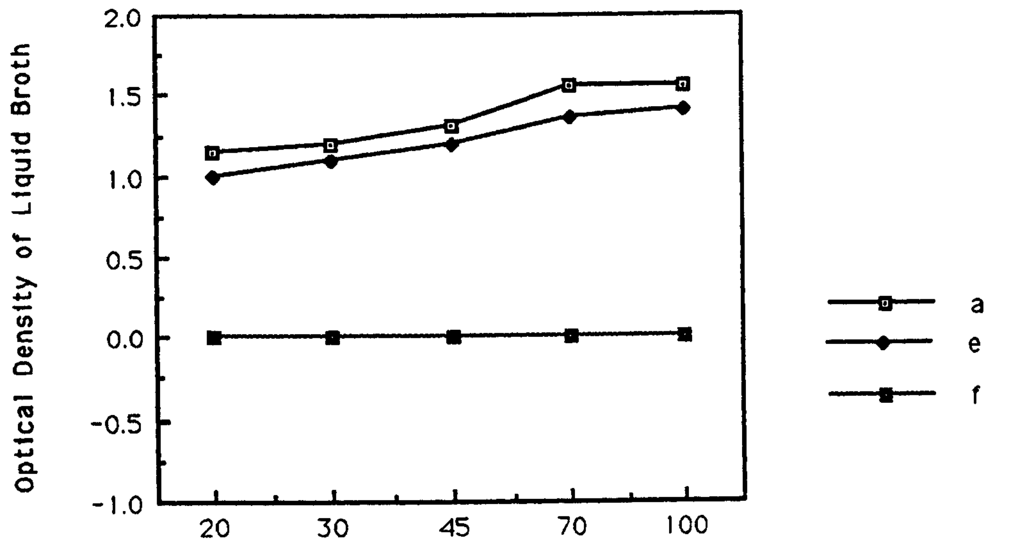

Figure 13 Effect of varying surface area for Ag/Pt-Coated Catheters Samples a, e, f.

Another invitro test is on the zone of inhibition which is the measurement of the zone of growth of a bacteria such as S. Aureus or E. Coli around the Ag and Ag/Pt coated catheters. The samples were put in liquid broth agar with $10^8$ cfu/ml E.Coli. The Table I below demonstrates the increased inhibition zone with the platinum-silver coating.

Table 1 Size of Inhibition Zone in mm.

| | E.Coli | S.Aureus |
|---|---|---|
| Teflon coated with Ag | 1 | 1 |
| Teflon coated with Ag/Pt | 2~4 | 2~3 |
| Latex coated with Ag | 1 | 1~2 |
| Latex coated with Ag/Pt | 2~4 | 1.5~3 |

Metals other than platinum (such as aluminum) have shown a marked increase in the inhibition zones demonstrating their potential use as an anti-microbial. Aluminum by it'self has little effect while both the aluminum and silver when used together have relatively large inhibition zones.

Invivo measurements with 3 New Zealand rabbits using Ag/Pt coated and uncoated vascular catheter inserted into a vein demonstrated the ability of the silver coatings to decease the rate of nasocormial infection [25].

In these measurements a bacterial challenge of S. Aureus at the catheter entry point was made [25, 26]. The results of the invivo and invitro experiment demonstrated the strong adhesion of the coating to the various sustrates.

### Conclusions

The invitro and invivo evaluations of silver and silver with other metals demonstrate that silver as a coating inside and outside of a catheter has great potential for blocking bacteria from entering the body along a catheter pathway.

In the above presentation silver has demonstrated it's value in a range of medical applications if used properly.

### Acknowledgements

R.B.B. acknowledge contribution of Charles Roman, Manju Shah, Sid Deliwala K.P. and R.B.B acknowledges partial support of Ben Franklin Partnerships of Southeastern Pennsylvania

### References

1. Beard, R. B., Hung, B. N. and Schmukler, R. *Annals of Biomedical Engineering,* **20**,1992, 395
2. Davidovitch, Z. et al, *Am. J. Orthod.* 77(No. 1), 1980
3. Korostoff, E. and Davidovitch, Z.,U.S Patent 4,153060
4. Beard, R. B., Hasan, S., Scoles, K. J., Onaral, B., U.S Patent 4,854,865
5. Scoles, K. J., Beard, R. B., Feng, D., Vanarsdall, R and Colby, G. T., *I.S.H.M.* 89 Proceedings, Baltimore, MD

6. Gedds, L.A., and Baker, L. E., *Applied Biomedical Instrumentation*, 3rd Ed, John Wiley 1989
7. Carim, H. M. U.S. Patent 4,377,170
8. Moody, G. J. and Thomas, J.O.R., *Selective Ion Sensitive Electrodes*, Merrow 1971
9. Roman, C. J., *M.S. Thesis in Biomedical Eng.* Drexel University, 1975
10. Shah, M., *M.S. Thesis in Biomedical Eng.*, Drexel University, 1981
11. Shah, M. Beard, R. B., Brownstein, M. and Niazy, N., *Proc. of Ninth Northeast Conf.*, Bioengineering Rutgers University, NJ 1981
12. Jan, Z., G.J. and Taniguchi, l l, *Chem. Rev.*, **53**, 1953
13. Bard, A.J. and Faulkner, L. R., *Electrochemical Methods*, John Wiley 1980
14. O'. M. Bockris, J. and Reddy, A.K.N., *Modern Electrochemistry* , Plenum Press, 1970
15. Valdes, L. B., *Proc., I. R.E.*, Feb 1954
16. Uhlir, A. Jr., *Bell Sys Tech. J.*, Jan 1955
17. Brownstein, M., *M.S. Thesis in Biomedical Eng.*, Drexel University, 1978
18. Pourbaix, M., *Atlas of Elctrochemical Equilibria in Aqueous Solutions*, 2nd English Ed 1974
19. Greene, M.D., Moebus, G.A., Baldwin, M.H., *Corrosion*, **29**(6), 1973
20. *First Intl. Conf. On Gold and Silver in Med.*, Bethesda, Md. 1987
21. Spadero, J. A., Chase, S.E. and Webster, D. A., *J Biomed. Mater. Res*, **20**, 1986
22. Sawyer, P. N., Ogonick, J. C., Boddy, P. J., *Surgery 61*, 1967
23. Croitoriu, et al, U.S. Patent 4,930,863
24. Shvets I., DeLaurentis, M, Beard, R. B., Pourreyzaei, K., *Trans 20 th Ann. Meeting Soc. For Biomaterials*, Boston, 1994
25. DeLaurentis, M, Shvets I., Beard, R. B., Pourreyzaei, K., *Trans 20 th Ann. Meeting Soc. For Biomaterials*, Boston, 1994
26 Pourreyzaei, K., Shvets I., DeLaurentis, Boxman, R. L., Beard, R. B., Croitoriu, N., Rastogi, R., Mukhtar, M., Logan, D. A., *Surface and Coatings Technology* (accepted for publication in special issue)

